# The role of leptin in the respiratory system: an overview

**DOI:** 10.1186/1465-9921-11-152

**Published:** 2010-10-31

**Authors:** Foteini Malli, Andriana I Papaioannou, Konstantinos I Gourgoulianis, Zoe Daniil

**Affiliations:** 1 Respiratory Medicine Department, University of Thessaly School of Medicine, University Hospital of Larissa, 41110, Greece

## Abstract

Since its cloning in 1994, leptin has emerged in the literature as a pleiotropic hormone whose actions extend from immune system homeostasis to reproduction and angiogenesis. Recent investigations have identified the lung as a leptin responsive and producing organ, while extensive research has been published concerning the role of leptin in the respiratory system. Animal studies have provided evidence indicating that leptin is a stimulant of ventilation, whereas researchers have proposed an important role for leptin in lung maturation and development. Studies further suggest a significant impact of leptin on specific respiratory diseases, including obstructive sleep apnoea-hypopnoea syndrome, asthma, COPD and lung cancer. However, as new investigations are under way, the picture is becoming more complex. The scope of this review is to decode the existing data concerning the actions of leptin in the lung and provide a detailed description of leptin's involvement in the most common disorders of the respiratory system.

## Introduction

In the past years, a growing number of studies have examined the potential role of leptin in the respiratory system. Accumulative data have identified foetal and adult lung tissue as leptin responsive and producing organs, while leptin's involvement in pulmonary homeostasis has become increasingly evident (Table 1). On the basis of this conception, researchers have sought to determine the impact of leptin on specific respiratory disorders, including obstructive sleep apnoea-hypopnoea syndrome (OSAHS), asthma, chronic obstructive pulmonary disease (COPD) and lung cancer. We review herein the current understanding on the actions of leptin in the lung, and summarize the recent advances on its role in the pathophysiology of respiratory diseases.

**Table 1:** Effects of leptin signaling in lung cells

Reference (year)	Effect	Comments
Vernooy et al^8 ^(2009)	Increased leptin and Ob-Rb expression in bronchial epithelial cells following smoke exposure Leptin induces phosphorylation of STAT-3 in NCI-H292 and A549 cell lines	Cells obtained from lung cancer patients who underwent lung surgery (disease free areas) A549 is a human alveolar epithelial cell line-NCI-H292 is a human bronchial epithelial cell line
Bruno et al^9 ^(2009)	Leptin increases cell proliferation and decreases TGF-β release in 16HBE cell line TGF-β decreases and fluticasone propionate increases leptin receptor expression in 16HBE cell line	16HBE is a human bronchial epithelial cell line
Nair et al^47 ^(2008)	Leptin inhibits PDGF-airway smooth muscle migration and proliferation and IL-13-induced eotaxin production	Cells obtained from lung cancer patients who underwent lung surgery (disease free areas)
Tsuchiya et al^49 ^(1999)	Leptin induces cell proliferation in SQ-5 cells by increasing the MAP kinase activity	SQ-5 is a clonal cell line derived from human lung squamous cell cancer

Abbreviations: STAT: signal transducers and activators of transcription, MAP: mitogen-activated protein, PDGF: platelet derived growth factor

### Leptin and the Leptin Receptor at a glance

Leptin, a 16KDa protein of 167 amino acids, represents the product of the *ob *gene which in humans is located on chromosome 7 [1]. The protein is synthesized and secreted mainly by white adipose tissue, apparently in proportion to fat stores, and thus is considered an adipokine [2]. However, leptin is produced in lower amounts by other tissues, such as the placenta [3], gastric fundic mucosa [4], and pancreas [5]. Regarding the lung, the *ob *gene is expressed in foetal lung tissue in baboons [6], and foetal rat lung fibroblasts [7] (Table 1). Interestingly, others have demonstrated the production of leptin in human peripheral lung tissue, namely bronchial epithelial cells, alveolar type II pneumocytes, and lung macrophages [8,9].

Accumulated evidence suggest that leptin production is mainly regulated by food intake; fasting reduces leptin levels while food consumption is associated with a transient increase in *ob *gene expression [10]. However, leptin levels can be influenced by other factors as well. Insulin and glucocorticoids can stimulate leptin secretion [11]. Leptin concentrations are increased during infection and sepsis [12], in accordance with the observation that leptin expression is up-regulated by various pro-inflammatory cytokines, including tumor necrosis factor-α (TNF-α) [13], interleukin-1 (IL-1) and leukaemia inhibitory factor [14]. In contrast to acute stimulation of the inflammatory system, chronic inflammation causes a reduction in leptin levels [15]. Moreover, the *ob *promoter is induced by several transcription factors, such as hypoxia inducible factor-1 (HIF-1) [16], and suppressed by others, like peroxisome proliferators-activated receptor-γ agonists [17]. Leptin expression is inhibited by testosterone, whereas it is increased by ovarian sex steroids [14] in agreement with the strong gender-related dimorphism of leptin levels (i.e. leptin is higher in females than age and body mass index (BMI) matched males) [12]. Finally, leptin concentrations are reduced by catecholamines [18].

The discovery of leptin was considered synonymous to the discovery of the antidote to obesity. *ob/ob *mice have a single base pair mutation in the leptin gene that results in the absence of functional leptin, increased body weight, hyperphagia, impaired energy homeostasis, and low resting metabolic rate. Exogenous administration of leptin reverses this phenotype [19]. Additional studies demonstrate that leptin crosses the blood brain barrier and serves as an afferent signal, originating from the adipose tissue, engaging distinct hypothalamic effector pathways to suppress appetite and augment energy expenditure [20]. However, in humans, the action of leptin as an anorexigen is more complex. Human obesity is associated with increased circulating leptin levels and a relative leptin "insensitivity" [21]. Central resistance to leptin might be the result of diminished brain leptin transport [22] and/or down-regulation of the leptin receptor in the central nervous system (CNS) [23].

Beyond its metabolic functions, leptin is implicated in various other physiologic processes, including the immune response, with effects in both innate and adaptive immunity. Indeed, leptin up-regulates the expression of several pro-inflammatory cytokines, such as TNF-α, IL-6, and IL-12, while it increases chemotaxis and natural killer cells function [24,25]. Leptin enhances T helper (Th) 1 response and suppresses Th2 pathways, whereas it can exert direct effects on CD4^+ ^T lymphocyte proliferation and macrophage phagocytosis [12,25,26]. Moreover, leptin stimulates the proliferative activity of human monocytes in vitro and up-regulates the expression of several activation markers, like CD25 and CD38 [24].

The pleiotropy of leptin is reflected by the multiplicity of its biologic effects in other tissues. Leptin increases sympathetic nervous system (SNS) activity [27,28], with possible implications in endothelial cell function and blood pressure homeostasis [29]. Furthermore, the adipokine up-regulates various pro-angiogenic factors, such as CC-chemokine ligand 2 (CCL2) [30], while synergistically stimulates angiogenesis with vascular endothelial growth factor (VEGF) [31], indicating that it may contribute to the promotion of neo-vascularization processes [32]. Additionally, leptin has been proposed to mediate wound re-epithelization and healing [33], bone turn-over and skeletal development [34], as well as fertility [35]. Moreover, data suggest that leptin stimulates insulin secretion, regulates fatty acid oxidation [36] and reduces cortisol synthesis [37]. The implication of leptin in lung physiology and pathophysiology is discussed extensively below.

The leptin receptor (Ob-R) is a member of the class I cytokine receptor super-family, which includes the receptors of IL-1, IL-2, IL-6 and growth hormone [38]. Alternate splicing of the leptin receptor gene (*db *gene) gives rise to six receptor isoforms that share a common extracellular and transmembrane domain, and a variable intracellular residue, characteristic for each type. The isoforms are classified according to the length of their cytoplasmic domain to four short (Ob-R_a_, Ob-R_c_, Ob-R_d _and Ob-R_f_) and one long form (Ob-R_b_), while a soluble form (Ob-R_e_) also exists [26]. The long functional isoform is expressed abundantly in the hypothalamus and is essential for signal transduction through Janus Kinase-signal transducer and activation of transcription factor (JAK-STAT) pathway [39]. The short isoforms are expressed in various tissues, such as the kidney, however their function has not been fully elucidated [38,40].

Importantly, *db *gene is expressed in lung tissue; studies in several animal models, including mice, rats, baboons and other animals, have identified Ob-R presence in the lung (Table 2) [6,7,40-46]. Interestingly, other studies have localized the expression of Ob-R in human airway smooth muscle cells [47], epithelial cells and submucosa of lung tissue obtained by bronchial biopsies [48]. Of great importance is the expression of Ob-R_b _in cells of the lung, like bronchial and alveolar epithelial cells, including type II pneumocytes [8,9,49]. Although the functional significance of the leptin receptors in the periphery is largely unknown, the existence of the functional receptor isoform indicates that the lung represents a target organ for leptin signaling.

**Table 2:** Lung cells as a source of leptin

Species	Cell type (source)	Reference
Baboon (foetal)	NA	[6]
Rat (foetal)	Fibroblasts	[7]
Human	Type II pneumocytes	[8]
Human	Lung macrophages	[8]
Human	Bronchial epithelial cells	[8,9]

Abbreviations: NA: Not applicable

### The role of leptin in lung development

Evidence indicate that leptin can be synthesized by foetal adipose tissue, and the placental trophoblast, while leptin and Ob-R genes are expressed in foetal lung tissue, thus suggesting its novel role in foetal lung growth and development (Table 2 and Table 3) [6,7,43,45]. Importantly, researchers have reported enhanced leptin production by foetal rat lung fibroblasts during the period of alveolar differentiation [7], while others have observed increased Ob-R abundance in foetal lung tissue in advanced gestation [6].

**Table 3:** Leptin Receptor expression in the lung

Species	Cell type	Isoform	Reference
Baboon (foetal)	Peripheral epithelial cells (including type II pneumocytes)	Ob-R_b _, Ob-R_s_	[6]
Human	SCLC cell line (H441)	NA	[7]
Rat (foetal)	Fibroblasts, type II pneumocytes	NA	[7]
Human	Bronchial epithelial cells/type II pneumocytes	Ob-R_b_	[8]
Human	Bronchial epithelial cells	NA	[9]
Mouse	NA	Ob-R_s_	[40]
Mouse	Peripheral bronchial/alveolar epithelial cells	NA	[41]
Calf	NA	Ob-R_b_	[42]
Mouse (foetal)	NA	NA	[43]
Mouse	NA	Ob-R_b_, Ob-R_a_, Ob-R_e_	[44]
Rabbit (foetal)	Type II pneumocytes	Ob-R_b_	[45]
Rabbit	NA	NA	[45]
Pig	NA	Ob-R_b_	[46]
Human	Airway smooth muscle cell	NA	[47]
Human	Epithelial cells/submucosa	NA	[48]
Human	NSCLC cell line (SQ-5)	Ob-R_b_	[49]

Abbreviations: Ob-R_s_: short isoforms, NSCLC: Non Small Cell Lung Cancer, SCLC: Small Cell Lung Cancer, NA: Not applicable,

Studies of several models of pulmonary development suggest a modulatory role for leptin in foetal lung maturity. Antenatal administration of leptin results in a significant increase of foetal rat lung weight, possibly due to an increase in the number and maturation of alveolar type II cells, accompanied by an induction in the expression of surfactant proteins B and C [50]. Interestingly, parathyroid hormone-related protein (PTHrP), an alveolar type II cell product that enhances type II cell differentiation, increases the production of leptin by lung lipofibroblasts [7,51]. Additionally, leptin stimulates surfactant protein synthesis when added to foetal rat lung explant culture [7,50], or foetal alveolar type II cell culture, thus suggesting the existence of a regulatory paracrine feedback loop in the foetal lung [45,51]. Further support is provided by studies demonstrating that cell stretch, known to stimulate the growth and differentiation of the alveolar septal wall, induces surfactant synthesis through enhancing the paracrine actions of leptin and PTHrP [51].

Accumulated evidence suggest a role for leptin in postnatal lung development. Interestingly, leptin concentrations on the seventh day of life are positively correlated with lung weight in neonatal lambs receiving leptin intravenously, suggesting its potential role in lung growth [52]. The pulmonary phenotype of genetically obese mice provides supporting evidence to the hypothesized implication of leptin in lung development; *ob/ob *mice exhibit significantly decreased lung volume and lower alveolar surface area at 2 weeks of age, when compared to heterozygotes or control animals [53].

Despite the remarkable power of the aforementioned observations, which suggest that leptin enhances lung maturation, the fact that they derive from animal lung development models represents a major limitation in extrapolating the results to the human species.

### Is leptin involved in Respiratory Control?

Studies in animal models have provided evidence indicating that leptin serves as a stimulant of ventilation. *ob/ob *mice exhibit increased breathing frequency, minute ventilation and tidal volume, associated with significantly elevated arterial P_a_CO_2 _and depressed hypercapnic ventilatory response (HCVR), present even before the onset of obesity, when compared to wild-type mice [54-57]. The aforementioned observations are evident during all sleep/wake states, although HCVR is more profoundly reduced during sleep [54]. Chronic leptin replacement restores the rapid breathing pattern and the diminished lung compliance associated with the obese phenotype [55]. To streamline these findings, leptin administration prevents weight gain in *ob/ob *mice, thus it is difficult to determine whether the attenuation of the respiratory complications is caused by mechanical factors or by a direct effect of leptin on lung growth and respiration [55]. However, acute leptin replacement results in a significant increase in baseline ventilation and chemosensitivity during sleep, independent of weight gain [54]. Importantly, leptin microinjections into the tractus nucleus solitarius in the brain of rats is associated with increased pulmonary ventilation and respiratory volume and enhanced bioelectrical activity of the inspiratory muscles suggesting that leptin may be implicated in ventilatory control through direct effects on respiratory control centres [58].

At this point it should be mentioned that the *ob/ob *model represents a model of obesity and systemic inflammation rather than a simple model of leptin deficiency with substantial diversities from human obesity that is associated with hyperleptinemia and central leptin resistance [59]. While clinical studies provide supporting evidence to the mouse-model observations indicating the critical role of leptin in ventilatory control (e.g. leptin is a predictor of lung function in various conditions, including asthma [60], heart failure [61] and is negatively correlated with lung volumes in COPD patients [62]) the pathophysiological significance of leptin regarding respiratory function in humans remains to be clarified.

## The role of leptin in diseases of the lung

Over the past years, extensive research has been conducted concerning the impact of leptin on various respiratory disorders. Mounting evidence have been published, as the picture is becoming more complex. The scope of this review is to decode the existing data and provide a detailed description of the involvement of leptin in the most common disease entities associated with the respiratory system.

### Obstructive sleep apnoea-hypopnoea syndrome (OSAHS) and obesity hypoventilation syndrome (OHS) (Table 4)

**Table 4:** The role of leptin in OSAHS and OHS

Reference (year)	Main message	Main limitations
Ip et al^68 ^(2000)	Leptin significantly correlated with AHI	Only males/Limited number of patients/Potential influence by comorbidities/No adjustment for FM
Campo et al^78 ^(2007)	Higher leptin is associated with reduced respiratory drive and reduced hypercapnic response	Conditions of blood sampling unknown/Potential influence by comorbidities
Philips et al^82 ^(2000)	Increased leptin in OSAHS	Only males/Limited number of patients/Low statistical power
Barcelo et al^86 ^(2005)	Decrease in leptin after nCPAP treatment in non-obese OSAHS	Only males/Limited number of patients/No adjustment for FM
Shimizu et al^90 ^(2002)	Significant decrease in leptin after 1 day of nCPAP The decrease of leptin correlated with cardiac sympathetic function	Only males/Limited number of patients/Potential influence by comorbidities Low statistical power
Phipps et al^96 ^(2002)	Leptin is a predictor for the presence of hypercapnia	Limited number of patients/Sex unknown

Abbreviations: FM: Fat Mass

OSAHS is a common disorder characterized by repeated episodes of partial or complete upper airway obstruction during sleep [63]. Approximately 90% of patients with OHS, a condition defined as a combination of obesity (i.e. BMI ≥ 30 Kg/m^2^) and sleep disordered breathing, have concurrent OSAHS (i.e. apnoea-hypopnoea index (AHI) > 5) [64], while 10-15% of patients with OSAHS develop hypoventilation and daytime hypercapnia [65].

Obesity is considered to be the most important risk factor of OSAHS [66]. The impact of obesity in sleep disordered breathing was originally reported to be mechanical but recent data suggest that adipose tissue can contribute to the genesis of the syndrome through its metabolic activity. The established role of leptin as a respiratory stimulant (discussed extensively above) raised the possibility that OSAHS may represent a leptin-deficient state. Inversely, several groups have demonstrated higher circulating leptin levels in OSAHS patients, when compared to age, sex, and weight-matched controls [67-72], while others have failed to document such a difference [73,74]. However, a collective comparison of these findings is difficult, since many of the aforementioned studies have included patients with comorbid conditions (e.g. arterial hypertension) that could serve as confounding factors [68,74]. The preceding data, exhibit substantial weakness originating from the relatively small number of subjects included and, additionally, the male predominance in the majority of these reports raises difficulties in extrapolating the results to the female sex.

In the light of these data, researchers have hypothesized that OSAHS is a leptin-resistant state, and that a relative deficiency in CNS leptin levels, due to an impaired transport across the blood-brain barrier, may induce hypoventilation, therefore contribute to the genesis of the syndrome [75-77]. Unfortunately, literature lacks data to confirm or to decline such a hypothesis, since, to our knowledge, no study until today has investigated leptin levels in cerebrospinal fluid (CSF) in OSAHS patients. Another explanation is an impairment in leptin activity in CNS, caused by down-regulation of central leptin receptors or defects in second messenger system [54,76-78]. Recently, researchers have identified a single nucleotide polymorphism in the leptin receptor gene associated with the presence of OSAHS [79]. This single amino acid change in the Ob-R molecule may result in altered signal transduction, generating a state of leptin resistance, in consistency with the latter hypothesis. However, others have failed to confirm an association of leptin and leptin receptor gene variations with the development of OSAHS [80], although the results should be interpreted with caution since the number of patients enrolled have been reported to be underpowered to detect a sufficient effect [81].

A subject of ongoing controversy is whether the presence of hyperleptinemia in OSAHS derives from adiposity or it reflects causality due to the effects of sleep-disordered breathing. Leptin levels are 50% higher in OSAHS patients than in controls, suggesting that other factors besides obesity contribute to the elevation of leptin [82]. In consistency with the previous results, leptin levels are significantly correlated with several indices of OSAHS severity, i.e. AHI, percentage of sleep time with less than 90% hemoglobin saturation (%T90), oxygen desaturation index, as well as with a variety of anthropometric measurements, including BMI, waist-to-hip ratio (WHR), and skinfold thickness [68-70,72,75,83,84]. However, the data derived are rather contradictive; some researchers have documented a significant positive correlation of leptin levels with AHI, even when controlled for BMI [70], while others have reported no significant correlation between leptin values after adjustment for BMI, WHR and waist circumference, with measures of disease severity, although WHR and T%90 were found to be the most significant variables in a model predicting leptin [69]. In keeping with the aforementioned concepts, other researchers have documented that BMI is the only parameter significantly and independently associated with leptin concentrations [83]. Similarly, other groups have reported that adiposity measures are the only predictive factors of leptin levels, while AHI was not found to be significant [75].

To make matters more complicated, studies have documented significantly higher leptin levels in non-obese OSAHS patients versus controls [85,86]. Data suggest that repeated sleep hypoxemia may promote leptin production independently of the degree of obesity. However, the authors provided evidence indicating that the location of the body fat deposition (e.g. visceral fat accumulation) may account for the increased leptin concentrations in non-obese OSAHS subjects [85]. Clearly, the aforementioned findings are inconclusive and due to their associative nature, cannot substantiate causality.

Additional studies examining the effects of nasal continuous positive airway pressure (nCPAP) treatment were designed to elucidate the exact association of leptin with OSAHS. Leptin levels decrease significantly in OSAHS patients, treated with nCPAP for a period of 3 days to 6 months, without any significant change in BMI observed [68,83,87-89]. The significant reduction in circulating leptin following 1 to 4 days of nCPAP therapy [87,90] suggests that OSAHS itself may stimulate, at least in part, leptin production independently of obesity. However, the mechanisms responsible are yet unclear, and no definite conclusions can be made since several groups have reported no significant changes in leptin levels after the application of nCPAP [91,92]. Interestingly, Barcelo et al [86] documented a marginal, yet significant, decrease in leptin levels associated with nCPAP treatment in non-obese OSAHS patients, while leptin concentrations were reported unchanged in obese subjects. Similarly, others have illustrated a more pronounced reduction of leptin levels in non-obese patients versus obese OSAHS patients [89]. The physiological explanation has not been fully elucidated, but data in the literature suggest that the decrease in leptin might be explained by the effect of treatment on sympathetic nerve activation [90], or may be associated with changes in haemodynamics and visceral blood flow [83]. Other possible explanations include the reduction in visceral fat accumulation and stress levels [93], or a reverse in the Ob-R sensitivity [94], consistent with the hypothesis of leptin resistance discussed above.

Few studies in the literature have examined the possible implication of leptin in OHS. As argued earlier, leptin deficient mice exhibit similar to OHS features, i.e. CO_2 _retention and depressed HCVR [95]. In obese patients, hyperleptinemia is associated with a reduction in respiratory drive and hypercapnic response, irrespective of anthropometric measurements [78], while circulating leptin is a predictor for the presence of hypercapnia [76,96]. Leptin concentrations are statistically significantly lower in OHS patients without OSAHS, when compared to BMI matched eucapnic obese subjects without OSAHS [97]. Additionally, the authors demonstrated a significant increase in leptin values following long-term non-invasive mechanical ventilation (NIVM), although the levels were still lower than those at the eucapnic group. Inversely, other researchers have reported a significant reduction in leptin levels in OHS patients receiving NIVM [98]. However, a direct comparison of these results can be misleading, since Yee et al [98] enrolled subjects with OHS associated with OSAHS. In contrast, others have reported higher circulating levels of leptin in OHS when compared to eucapnic obese subjects despite similar degree of body fat [96]. Serum leptin served as a predictor for the presence of hypercapnia, suggesting that higher and not lower leptin levels predisposes to OHS. However, this study included patients with concurrent OSAHS that could serve as a confounding factor. In the light of these data, some have raised the possibility that OHS may be characterized by a more profound degree of leptin resistance than OSAHS, although this hypothesis requires further validation by more extensive studies [93].

### Chronic Obstructive Pulmonary Disease (COPD) (Table 5)

**Table 5:** The role of leptin in COPD

Mechanism studied	Reference (year)	Main message	Main limitations
Cachexia-stable COPD	Takabatake et al^102^ (1999)	Leptin production regulated physiologically and not correlated with TNF-α or sTNF-R	Only males/Limited number of patients/No adjustment for FM
	Takabatake et al^104 ^(2001)	Absence of circadian rhythm of leptin	Only males/Limited number of patients
	Schols et al^106 ^(1999)	Leptin related to sTNF-R55 in emphysema	Only males/Limited number of patients/Patients received CS
Exacerbation	Creutzberg et al^108 ^(2000)	Increased leptin (serial measurements) Leptin positively correlated with sTNFR-55	Limited number of patients/Patients with hospital stay < 7 days excluded/Patients received CS/Only severe COPD
	Kythreotis et al^109 ^(2009)	Leptin positively correlated with TNF-α	Patients received CS

Abbreviations: FM: fat mass, CS: corticosteroids

COPD is a disease state characterized by airflow limitation that is not fully reversible, usually progressive, and associated with an abnormal inflammatory response of the lung to noxious particles or gases [99]. Researchers have speculated that a potential link between obesity and COPD subsists since low BMI and weight loss is associated with increased mortality in patients suffering from COPD [100]. However, the mechanisms underlying this association are not yet fully elucidated.

Studies in the literature have examined the hypothesis that underlying abnormalities in the leptin feedback mechanism might be involved in the impaired energy balance responsible for the cachexic status and muscle wasting commonly seen in COPD [101]. However, researchers have failed to demonstrate the presence of inappropriately increased leptin levels in cachexic stable COPD patients [102,103], while there is no statistically significant relationship detected between circulating leptin and the activated TNF-α system [102-105]. In contrast, others have reported a significant partial correlation coefficient between leptin and soluble tumour necrosis factor receptor 55 (sTNF-R55), when adjusted for fat mass (FM) and oral corticosteroid use in the emphysematous subtype of COPD, but not in chronic bronchitis patients, while leptin levels were associated with FM in line with the reported feedback mechanism involved in the regulation of body weight [106]. Although leptin seems to be regulated physiologically, low leptin levels may contribute to sexual disturbances, impaired glucose tolerance, and higher frequency of pulmonary infection, observed in COPD patients [102], while leptin has been associated with the presence of osteoporosis in COPD subjects [62]. To gain a more comprehensive understanding, Takabatake et al [104] examined the circadian rhythm of circulating leptin in COPD and documented its absence in cachexic COPD patients, while it was preserved in normal weight COPD subjects. Interestingly, the very low frequency component of heart rate variability, which has been considered to reflect neuroendocrine and thermoregulatory influences to the heart, showed similar diurnal rhythm with circulating leptin in all study groups [104]. These data suggest that the loss of the physiologic pattern of leptin release may have clinical importance in the pathophysiologic features in cachexic patients with COPD, such as abnormalities of the autonomous nervous system and the hypothalamic-pituitary axes, or may represent a compensatory mechanism to maintain body fat content [104].

Researchers have investigated the possible involvement of leptin during the acute exacerbations of COPD. Malnourished patients experiencing exacerbation, exhibit significantly higher leptin levels, compared to normal-weight stable COPD patients, an observation not replicated when compared to malnourished stable COPD patients [107]. Similar results have been reported by other groups [103]. Importantly, leptin values, corrected for FM, are significantly elevated in COPD patients during acute exacerbation versus controls [108,109]. Leptin concentrations gradually decrease throughout the exacerbation, but when corrected for FM, remain significantly elevated during hospitalization [108,109]. The normal feedback regulation of leptin by FM is preserved on Day 7 of the exacerbation, although dissociation has been reported on Day 1, possibly due to a temporary dysfunction related to the event [108]. The natural logarithm (LN) of leptin is inversely correlated with the dietary intake/resting energy expenditure index (indicating the role of leptin in energy balance) and positively correlated with sTNF-R55 (after correction for FM) [108]. Other researchers have reported a positive correlation between TNF-α and leptin on Day 1 of admission [109]. sTNF-R55 significantly explains 66% of the variation in energy balance in Day 7 of the exacerbation, while leptin is excluded, suggesting that the influence of leptin is under the control of the systemic inflammatory response [108].

The airflow limitation in COPD is linked to structural changes, including the presence of an abnormal inflammatory pattern detected in each lung compartment [110]. AKR/J mice (i.e. a strain that presents similar to COPD anatomic abnormalities following cigarette smoke exposure for 4 months) exhibit reduced Ob-R expression in the airway wall, upon smoke exposure [111]. Inversely, stimulation of bronchial epithelial cells and alveolar type II pneumocytes, isolated from human lung tissue, with increasing doses of cigarette smoke condensate results in a significant induction of leptin and Ob-R_b _m-RNA, suggesting that smoking itself may increase the expression of the leptin/leptin receptor system in lung tissue [8]. However, others have demonstrated down-regulation of leptin/leptin receptor system in bronchial epithelial cells of proximal airways of mild-to-severe COPD patients, when compared to tissues obtained from non-smoking subjects [48], while immunohistochemical studies show that leptin expression is increased in bronchial epithelial cells and alveolar macrophages in the peripheral lung of COPD patients (GOLD stage 4) [8]. Additionally, leptin is over-expressed in the submucosa of proximal airways of COPD patients [48]. The diversities observed in pulmonary leptin/leptin-receptor system expression among COPD patients, symptomatic smokers and never-smokers despite similar anthropometric measurements, lend further support to the concept of local production of leptin in the lung [8].

Accumulated evidence suggest that leptin may be involved in the local inflammatory response seen in the airways of COPD patients, hypothetically regulating the infiltration and the survival of inflammatory cells in the submucosa of COPD patients [48]. Interestingly, leptin's up-regulation in the proximal airways correlates to the expression of activated T lymphocytes (mainly CD8^+^) and to the absence of apoptotic T cells [48]. In addition, leptin is detected in induced sputum of patients with COPD, whereas it is significantly positively correlated with inflammatory markers measured in induced sputum, such as CRP and TNF-α [112]. Importantly, plasma and sputum leptin levels are inversely correlated. In harmony with the previous results, the presence of Ob-R_b _in lung epithelium and inflammatory cells combined with the fact that the lung is a source of leptin, suggests the existence of a paracrine cross-talk between resident pulmonary epithelial cells and immune cells in response to noxious particles [8]. This hypothesis needs further validation by subsequent studies, enrolling a larger number of patients and including experiments that will shed further light to the pathophysiological role of leptin in the pathogenesis of COPD.

Recently, researchers have reported that COPD patients carrying minor alleles of polymorphisms in the Ob-R gene are less susceptible to loss of lung function, as indicated by %FEV_1 _decline [111]. Although the functional significance is not known, these data have led to the hypothesis that the Ob-R gene may serve as a novel candidate gene for COPD.

### Asthma (Table 6)

**Table 6:** The role of leptin in asthma

Mechanism studied	Reference (year)	Main message	Main limitations
Structural changes	Bruno et al^9 ^(2009)	Leptin/leptin receptor expression in bronchial epithelial cells is reduced in mild uncontrolled and severe asthma	Limited number of patients/Patients treated with corticosteroids
Animal studies	Shore et al^117 ^(2003)	Increased response to ozone in *ob/ob *mice	*ob/ob *mice exhibit low lung size (potential mechanical bias)
	Luet et al^119 ^(2006)	Increased responses to ozone in *db/db *mice	*db/db *mice exhibit low lung size (potential mechanical bias)/Only female mice
	Johnston et al^121 ^(2008)	Mice with diet-induced obesity exhibit innate AHR	Control mice were overweight
	Shore et al^127 ^(2005)	Enhanced metacholine responsiveness in leptin-treated mice	Clinical relevance unknown
Clinical studies	Guler et al^124 ^(2004)	Leptin is a predictive factor for childhood asthma	No adjustment for FM/Lack of correlation of leptin with PFT
	Sood et al^126 ^(2006)	Higher leptin in asthmatics	Asthma diagnosis based on self-questionnaire/No adjustment for FM

Abbreviations: AHR: airway hyper-responsiveness, FM: fat mass, PFT: pulmonary function testing

Asthma represents a chronic inflammatory disorder of the airways associated with airway hyper-responsiveness that leads to recurrent episodes of widespread, and often reversible, airflow obstruction within the lung [113]. Obesity is a risk factor for asthma, while studies indicate that adiposity may increase disease severity in asthmatic subjects and possibly alter the efficacy of standard asthma medications [114-116]. The mechanisms underlying the relationship between obesity and asthma have not been fully established yet, however, experimental evidence suggests that changes in adipose-tissue derived hormones, including leptin, as well as other factors, are possibly implicated.

*ob/ob *mice exhibit significantly elevated pulmonary resistance (R_L_) and responsiveness to metacholine in baseline conditions, while ozone (O_3_) exposure results in greater increase in these two parameters, associated with an enhanced expression of bronchoalveolar alveolar lavage fluid (BALF) protein, eotaxin, and IL-6 when compared to lean controls [117]. Acute leptin replacement in chronically leptin-deficient mice cannot reverse the enhanced inflammatory response. However, mice fasted overnight exhibit reduced leptin levels, associated with a significant increase in R_L _and airway responsiveness following O_3 _exposure, as compared to fed mice [118]. The restoration of leptin to fed levels prevented the fasting induced changes in response to O_3_. Exogenous leptin administration in wild-type mice results in increased O_3_-induced cytokine and protein release into BALF [117]. Similarly to the *ob *murine model, *db/db *mice (i.e. mice that lack functional Ob-R_b _isoform due to a mutation in the cytoplasmic domain of the receptor) and carboxypeptidase E-deficient (CPE^fat^) mice (i.e. a strain characterized by obesity, resulting from a functional mutation in the gene encoding carboxypeptidase, and increased leptin levels) present increased baseline airway responsiveness, as well as augmented responses to O_3 _exposure, when compared to their lean controls [119,120]. In harmony with the latter results, mice with diet-induced obesity exhibit innate AHR and enhanced O_3_-induced pulmonary inflammation, similar to that observed in genetically obese mice [121]. Collectively, the aforementioned findings suggest that leptin may have the potential to augment the pulmonary response to acute O_3 _exposure, but other effects of obesity may also play an important role [122]. Since innate AHR is a common feature of leptin and leptin receptor deficient mice, as well as CPE^fat ^mice and mice with diet induced obesity (i.e. mice with reduced and mice with increased leptin concentrations) it seems unlikely that the adipokine can act as an intermediary in the causal pathway [122].

Clinical studies provide confounding evidence to the mouse-model observation regarding the role of leptin in asthma. Overweight asthmatic children present twice as high leptin levels as those without asthma, despite no differences in BMI [123]. Similar results are documented by other researchers; asthmatic children, especially asthmatic boys, exhibit higher leptin levels compared to controls [124]. Leptin concentrations are significantly associated with bronchodilator response in overweight/obese men, but not in overweight/obese women [125]. Furthermore, leptin levels, even when adjusted for BMI, are predictive of asthma in male subjects [124]. Additionally, increased BMI and leptin concentrations are associated with asthma in adults, but when adjusted for leptin, no effect is observed in the association among BMI and asthma, indicating that the association is not mediated by the leptin pathway alone [126]. In contrast, others have failed to document any direct association between leptin and the presence of asthma [60].

Increasing evidence suggest that the pro-inflammatory effects of leptin may contribute to the higher incidence of asthma in the obese population. As discussed previously, administration of leptin to wild-type mice enhances O_3_-induced airway inflammation [117], while ovalbumin sensitization and challenge increases serum leptin levels in mice [127]. Additionally, in animal models, exogenous leptin enhances the phagocytosis by macrophages and the production of TNF-a, IL-6 and IL-12 [124]. Administration of pro-inflammatory cytokines, such as TNF-α and IL-1, in mice results in a dose-dependent increase in leptin concentrations [126]. However, since these cytokines have been implicated in the pathophysiology of asthma [124] it is conceivable that the disease-related inflammation induces the release of leptin from the adipose tissue or the lung itself, which may in turn increase airway inflammation and hyper-responsiveness through a continuous interaction [122,126,128].

Over the past few years, researchers have hypothesized that decreased immunological tolerance, as a consequence of immunological changes induced by adipokines, may be implicated in the pathogenesis of allergic asthma [129]. As argued above, leptin-treated animals exhibit augmented responses to metacholine and increased levels of IgE, following ovalbumin challenge, when compared to saline-infused mice [127]. No difference on the inflammatory response in the airways was observed between the two study groups. In keeping with the aforementioned results, leptin and IgE levels are significantly correlated in asthmatic children [124]. Interestingly, atopic asthmatic boys have significantly higher leptin levels than non-atopic asthmatic subjects. Additionally, in vitro studies have documented that leptin can significantly up-regulate the cell surface expression of intracellular adhesion molecule (ICAM)-1 and CD18 and suppress those of ICAM-3 and L-selectin in eosinophils [130], while it augments alveolar macrophage leukotriene synthesis [131]. The latter results suggest that leptin may induce accumulation of eosinophils and may enhance inflammatory processes at sites such as the lung or the airways, and thereby augment allergic airway responses, at least in part [130,131].

Additionally, studies have raised the issue whether leptin may play an important role on asthma pathophysiology through its ability to activate SNS. Leptin increases the activity of the adrenal medulla and sympathetic nerves in various organs, although its impact on the sympathetic nerves of the lung is unknown [132,133]. On the basis of this conception, researchers have examined the effects of leptin on human airway smooth muscle cells and airway remodeling associated with asthma; leptin itself cannot promote muscle proliferation, migration or cytokine synthesis, suggesting that the effects of obesity on asthma may not be attributed to a direct effect of leptin on airway smooth muscle [47]. Leptin has no proliferative effect when administered in a human airway smooth muscle cell line culture, although it stimulates the release of VEGF by these cells [134]. However, the expression of leptin/leptin receptor in bronchial epithelial cells is significantly reduced in patients with mild uncontrolled asthma and severe treated asthma versus patients with mild controlled treated asthma and healthy volunteers, while leptin and leptin receptor expression are inversely correlated with reticular basement membrane thickness suggesting that leptin/leptin receptor expression may be associated with the airway remodeling observed in asthma, implicating the adipokine in the homeostasis of lung tissue [9].

### Lung Cancer (Table 7)

**Table 7:** The role of leptin in lung cancer

Reference (year)	Main messages	Main limitations
Ribeiro et al^138 ^(2006)	Polymorphism in the promoter of leptin gene associated with increased risk for NSCLC	Controls younger than patient group/Smoking status of controls unknown
Aleman et al^142 ^(2002)	Lower leptin in NSCLC vs controls	No adjustment for FM/Only advanced stage disease
Karapanagiotou et al^146 ^(2008)	No association of leptin to histological type, differentiation grade, disease stage, survival or time to disease progression	Controls and patients not age and sex matched/ Only advanced stage disease
Carpagnano et al^147 ^(2007)	Higher leptin in NSCLC vs controls	No adjustment for FM/Limited number o f patients/Non-advanced disease stage

Abbreviations: NSCLC: Non small cell lung cancer, FM: Fat mass

Increased BMI is significantly associated with higher death rates due to cancer [135], and it is well established that obesity increases the risk of cancer developing in numerous sites [136,137]. Can leptin be the mediator linking obesity with cancer?

A functional polymorphism in the promoter region of leptin gene is associated with a threefold increased risk of developing non-small cell lung cancer (NSCLC) [138]. The over-expressing variant is associated with earlier onset of lung cancer, but not with advanced metastatic disease, suggesting that continuous exposure to higher leptin concentrations due to the polymorphism in the leptin gene may accelerate cancer initiation [138]. This hypothesis is further strengthened by other groups who observed increased leptin levels in NSCLC patients and recognized leptin as a risk factor for cancer, even after controlling for BMI and recent weight loss [139].

In accordance with the previous studies, primary cultures of tracheal epithelial cells of *db/db *mice demonstrate significantly lower cell proliferation versus those of their lean litternates, while administration of leptin significantly increased cell proliferative ability in lean mice, but not in *db/db *mice [49]. Leptin has a stimulatory action on a clonal cell line derived from human lung squamous cell cancer (SQ5 cells), an effect mediated through mitogen activated protein (MAP) kinase activity, indicating that leptin may act as a growth factor. On the contrary, in an experimental pulmonary metastasis model, *ob/ob *and *db/db *mice present a remarkably increased number of metastatic colonies when compared to wild-type mice [140]. Administration of leptin in *ob/ob *mice abolished the increase in metastasis, indicating a rather prophylactic role of leptin. However, when cancer cells were inoculated orthotopically, through a chest incision, tumor growth at the implanted site was comparable among the groups.

Studies have led to the hypothesis that leptin contributes in cancer development, at least in part, through its up-regulatory role in the inflammatory system [141]. Leptin affects both innate and adaptive immunity by stimulating and activating neutrophils, macrophages, blood mononuclear cells, dendritic cells and T cells, and consecutively their products, which may induce chronic inflammation and lung carcinogenesis [141]. However, until today, this complex interplay between leptin, immune system, and cancer has received only some experimental support and further investigations are required.

A number of studies have examined the possible role of leptin in the pathogenesis of cancer-related weight loss. In consistency with earlier studies [142-145], Karapanagiotou et al [146], reported no differences in serum leptin levels, adjusted for sex and BMI, among advanced NSCLC patients and healthy controls. Leptin levels did not correlate with the histological type, differentiation grade, disease stage, overall survival, or time to disease progression, and there were no differences presented between patients with and without weight loss. Therefore, leptin cannot serve as a diagnostic or prognostic factor in advanced NSCLC. Moreover, these results suggest that cancer anorexia and cachexia are not due to a dysregulation of leptin production. The aforementioned observations are in contrast with those reported by other researchers, who observed higher concentrations of leptin in NSCLC patients *vs*. controls [147]. Patients recruited in the latter study had mainly non-advanced disease and there was no adjustment of leptin levels for FM, factors that can attribute to the discrepancies among studies.

### Infectious diseases of the lung (Table 8)

**Table 8:** The role of leptin in infectious diseases of the lung

Infectious disease	Reference (year)	Main message	Main limitations
Pneumonia	Mancuso et al^150 ^(2002)	*Klebsiella pneumonia *administration results in increased leptin (WT mice) and increased mortality (*ob/ob *mice)	Experimental condition not well corresponding with clinical pneumonia/Only female mice
	Hsu et al^152 ^(2007)	Increased mortality following pneumonococcal pneumonia (*ob/ob *mice) Leptin administration improves survival	Experimental conditions not well corresponding with clinical pneumonia/Only female mice
	Diez et al^155 ^(2008)	No differences in leptin in pneumonia vs controls Leptin lacks prognostic value for pneumonia lethality	Possible influence by comorbidities/Only hospitalized patients included
Tuberculosis	Buyukoglan et al^159 ^(2007)	Lower leptin in tuberculosis	No adjustment for FM/Higher BMI in controls/Limited number of patients
	van Crevel et al^161 ^(2002)	Leptin increases during antituberculous treatment	No adjustment for FM
	Cakir et al^163 ^(1999)	Higher leptin in tuberculosis No significant difference in leptin before and after antituberculous treatment	No adjustment for FM/Limited number of patients

Abbreviations: WT: wild-type, FM: fat mass,

#### Pneumonia

Recently, several reports have identified a role for leptin in regulating immune function [24,25] while leptin levels acutely increase during inflammation, infection and sepsis [12]. Furthermore, leptin deficiency has been associated with an increased frequency of infection [148,149]. Interestingly, leptin levels in serum, BALF and whole lung homogenates are elevated in wild-type mice, following intra-tracheal challenge with *Klebsiella pneumoniae *[150]. Additionally, *ob/ob *mice exhibit increased susceptibility and enhanced lethality following *K. pneumoniae *administration, as compared to wild-type mice, associated with impaired macrophage and neutrophil phagocytosis of the microorganism, and reduced macrophage leukotriene synthesis in vitro [150,151].

Concerning the impact of chronic leptin deficiency on gram-positive pneumonia, o*b*/*ob *mice display reduced survival following intra-tracheal challenge with *Streptococcus pneumoniae *[152]. This impairment is associated with increased pulmonary cytokine and lipid mediator levels, and defective alveolar macrophage phagocytosis and neutrophil polymononuclear (PMN) leukocyte killing in vitro. However, leptin administration to *ob/ob *mice in vivo improved pulmonary bacterial clearance and survival [152]. Furthermore, a physiologic reduction in leptin, induced by acute starvation, in a murine model of pneumococcal pneumonia, was associated with reduced PMN accumulation, IL-6 and macrophage inflammatory protein (MIP)-2 levels in BALF, impairment of leukotriene B_4 _(LTB_4_) synthesis and phagocytosis, and killing of *S. pneumoniae *in vitro [153]. Leptin administration to fasted mice corrects these defects. In contrast, others have failed to detect differences concerning the extent and severity of lung inflammation, and the bacterial outgrowth in the lung, during either gram-positive or gram-negative pneumonia, in *ob/ob *or wild-type mice [154].

To gain a more comprehensive understanding concerning the role of leptin in human lung infections, Diez et al [155], compared leptin levels in patients hospitalized for community acquired pneumonia and healthy controls and reported no significant differences in the two study groups, after adjusting for BMI, whereas leptin was inversely correlated with inflammatory markers. Interestingly, patients who died exhibited significantly lower leptin levels *vs*. controls. One of the most remarkable ascertainments of this study was the observation that leptin lacked independent prognostic value, since it was displaced by nutritional status on multiple logistic regression analysis, suggesting that leptin cannot act as an inflammatory reactant, but as a nutritional marker. Collectively, the few data that are present in the literature need further validation before any definite conclusions can be made.

#### Tuberculosis (TB)

Obesity is associated with lower risk of pulmonary TB [156]. Thin subjects are more likely to develop active TB and this may be a result of a relative deficiency of leptin [157,158]. A number of studies report that leptin levels are suppressed in tuberculous patients versus controls [159-161]. TB associated reductions of leptin are mediated independently by weight loss and prolonged inflammation [161], while leptin cannot account for the weight loss and anorexia associated with the disease [162]. Interestingly, leptin levels show no significant difference, corrected for energy balance and FM, at baseline and after TB treatment, in all but one study [159,161,162]. One may hypothesize that the prolonged inflammatory response in TB down-regulates or exhausts leptin production [161]. Since leptin is important for cell mediated immunity, low leptin concentrations during active TB may contribute to increased infection susceptibility, disease severity, and recovery with sequelae lesions [159,161]. However, the reduction of leptin levels may represent a protective component of the immune response in pulmonary TB [159]. The previous findings are not replicated in two studies, which report higher leptin concentrations in patients with active pulmonary TB versus controls [163,164].

Evidence in the literature demonstrates the presence of lower pleural fluid leptin levels in tuberculous pleural effusions when compared to other exudates [160,165]. Pleural fluid leptin levels may be used for the diagnosis of tuberculous pleural effusions (sensitivity 82,1%, specificity 82,4% for cut-off value of 9,85 ng/ml), however, the diagnostic value of low pleural fluid leptin was not as good as that of conventional methods, like adenosine deaminase [160].

Data from animal models suggest that leptin plays a role in the early immune response to pulmonary infection with *Mycobacterium tuberculosis*, most likely by mediating an effective interferon-γ driven Th1 response, adequate lymphocyte trafficking and granuloma formation [166]. *ob/ob *mice intra-nasally infected with live virulent *M. tuberculosis *display a transiently reduced host defense that is partially restored after leptin replacement [166]. Additionally, leptin deficient mice exhibit delayed mycobacterial elimination when challenged with high-dose aerosol of *Mycobacterium asbcessus*, when compared to wild-type controls [167]. Clearly, the latter hypothesis needs further confirmation from clinical studies.

### Acute Lung Injury (ALI)

Most of our knowledge regarding the role of leptin in ALI results from experimental animal models studies. The few data available are rather controversial and inconclusive. Researchers have demonstrated that *db/db *mice develop less edema and injury, whereas exhibit lower mortality in response to hyperoxia, when compared to control animals [41]. In addition, intratracheal instillation of leptin produces lung edema in wild-type mice, but not in *db/db *mice, suggesting that leptin induces ALI-related changes [41]. In contrast, exogenous leptin administration in rats significantly abrogates ALI and reduces mortality in cerulein-induced acute pancreatitis [168]. To make matters more complicated, *ob/ob *mice exhibit increased resistance to hyperoxia-induced ALI, when compared to control animals, while leptin or anti-leptin antibody administration to wild-type mice has no effect on the course of the hyperoxic injury [169]. The latter findings suggest that the adipokine itself does not play an essential role in oxygen-induced alveolar injury [169]. More studies are definitely required to assess the implication of leptin in ALI.

### Diffuse Parenchymal Lung Diseases (DPLDs)

To our knowledge, there are no studies in the literature examining the association of leptin with DPLDs. Investigations should be designed in order to examine the possible role of leptin on DPLDs pathogenesis.

## Conclusions

The role of leptin in lung physiology and pathophysiology has been studied extensively in the last few years. Undoubtedly, leptin has emerged in the literature as a multifunctional hormone with versatile activities and complex counteractions with other cytokines and adipokines. However, decoding its pulmonary impact is not an easy task, since the role of leptin cannot always be separated from obesity and the biology of adipose tissue. Currently, the effect of leptin signaling in the respiratory system remains controversial, possibly due to the fact that much of the existing knowledge derives from animal models of obesity (e.g. *ob/ob *model) that cannot identically represent the complex biological state of human obesity.

The presence of the functional leptin receptor in the lung recognizes the potential involvement of leptin in the pathogenesis of respiratory disorders, however, whether it represents a friend or a foe is not yet elucidated. Although animal studies provide direct indications that leptin enhances lung maturation and stimulates ventilation, further clinical studies are warranted in order to evaluate its significance in humans. The increased leptin levels observed in OSAHS cannot exclude the possible involvement of leptin in the depressed respiratory response during sleep since studies have not yet examined whether the disease is a leptin-resistant state, like obesity per se. Research data suggest that leptin/leptin receptor system expression and signaling is altered in the airways of patients with asthma and COPD. However, whether it represents an epiphenomenon or a pathogenetic mechanism remains poorly defined, revealing the need for further basic research studies. As for lung cancer, the role of leptin as a growth factor, derived by data examining the effects of leptin in cell lines, requires validation by experimental studies examining its pathophysiological impact on cancer development.

The boundary from research to clinical application is far from being crossed, as the current data have not revealed an exact role for leptin in the diagnosis, management or follow-up of patients with diseases of the respiratory system. As new investigations are under way, additional consequences of the action of leptin will emerge, adding more information to the already large body of knowledge and thus provide, possibly unexpected, answers to the questions that remain to be answered to date.

## Competing interests

The authors declare that they have no competing interests.

## Authors' contributions

FM, ZD and AP were involved in the study conception and design. FM performed the acquisition and interpretation of data and prepared the manuscript. ZD and KG were involved in revising the manuscript for important intellectual content. All authors read and approved the final manuscript.
